# Effect of Group Contingency Type on Walking: Comparisons of Effectiveness and Cost Efficiency

**DOI:** 10.3389/fpsyg.2021.655663

**Published:** 2021-05-28

**Authors:** Heewon Kim, Changseok Lee, Seoi Lee, Kyong-Mee Chung

**Affiliations:** Department of Psychology, Yonsei University, Seoul, South Korea

**Keywords:** contingency management, group contingency, mHealth, mobile device, walking

## Abstract

Group contingency (GC) is an effective and cost-efficient strategy that can be successfully applied to technology-based interventions. This study examined the relative effectiveness and cost efficiency of three types of technology-based group contingencies on walking among adults. Seventy two students were divided into teams of three. Each team was randomly assigned to one of three GC conditions (independent, interdependent, or dependent) and underwent 66 days of technology-based group contingency intervention. Sixty five participants completed the intervention and 61 completed the follow-up assessment 2 months later. Step counts and self-reported walking activity increased after the intervention under all three conditions. The proportion of participants that met the target step counts was significantly higher under the dependent group contingency condition. However, 2 months later, intervention effects were not maintained under any condition. For cost efficiency, the increase in step count per point was significantly higher under the interdependent group contingency condition. Group cohesion and social validity (point satisfaction and point utility) were significantly higher under the dependent group contingency condition. Finally, the clinical implications and limitations of this study are discussed.

## Introduction

Regular physical activity is an essential component of a healthy life (Oja et al., 2010). Not only does it lower the risk of major physical illnesses, such as obesity (Jakicic, 2009; Petridou et al., 2019), heart disease (Sattelmair et al., 2011; Fiuza-Luces et al., 2018), and diabetes (Jeon et al., 2007; Najafipour et al., 2017), it is also beneficial for mental health because it alleviates depression and anxiety (Ströhle et al., 2005; Rethorst and Trivedi, 2013; Sadeghi Bahmani et al., 2019). In particular, walking is commonly recommended for physical activity (Heron et al., 2014), because it is cost-free and requires no additional training (Tudor-Locke and Bassett, 2004; Tudor-Locke et al., 2009), and its intensity can easily be modified (Morris and Hardman, 1997). However, recent studies have reported physical inactivity among adults, with most engaging in less than half of the recommended level of walking activity per day (Bassett et al., 2010). Learning theory explains this noncompliance *via* the immediacy of reinforcement; whereas the costs of physical activity, such as fatigue, are experienced immediately, the benefits, such as losing weight, are delayed (Martin et al., 2012; Mitchell et al., 2020).

With the recent growth in mobile and Internet technologies, new strategies are being proposed to promote physical activity (Direito et al., 2017). Among these, mobile health (mHealth) is drawing increased attention (WHO, 2011; Glynn et al., 2014), as it provides cost-effective and easily distributed health management services. Mobile applications are the most accessible form of mHealth, and an analytical report found that the rate of health and fitness application usage had more than tripled in 3 years, with most of the applications related to physical activity (Kesiraju and Vogels, 2017). However, mHealth applications have frequently been criticized for the following two reasons (Mateo et al., 2015): first, theories in behavioral change are rarely considered during the planning and development phases of these applications; and second, testing their effectiveness before commercialization is also often bypassed. Thus, many researchers have highlighted the need to supplement these applications through strategies based on theories of effective behavioral change (Fanning et al., 2012; Coughlin and Stewart, 2016; Walsh et al., 2016).

Contingency management (CM) is a strategy based on the principles of operant conditioning, which increases the frequency of a target behavior in the future by providing a specific reinforcer (e.g., voucher, cash, lottery, etc.) upon the occurrence of the target behavior. CM has typically been applied in people, and its effectiveness has been measured in terms of promoting physical activity across different age groups, including children (Epstein et al., 2004), adults (Cohen et al., 2007; Washington et al., 2014), the elderly (Matteson, 1989), and in clinical groups, such as people with obesity or a physical disease (Petry et al., 2011; Byrne et al., 2012). Recently, CM has been actively applied in mHealth as an intervention strategy to monitor behaviors of an individual in real-time using mobile and Internet technologies, and to remotely deliver the reinforcer immediately upon occurrence of the behavior. This strategy is referred to as technology-based CM (Asch et al., 2012; Dallery et al., 2015a). Technology-based CM is cost efficient, as it reduces costs for labor and facility maintenance (King et al., 2013; Kurti and Dallery, 2014). Additionally, technology-based CM can be provided to several people simultaneously, including those with limited accessibility to treatment facilities because of physical distance and socioeconomic status (Kurti and Dallery, 2014; Dallery et al., 2019). In response, researchers have adopted various CM strategies (e.g., intermittent reinforcement, lottery incentives, etc.) to promote physical activity, and the effectiveness of technology-based CM was found to be similar or superior to that of traditional, in-person CM (Kurti and Dallery, 2013; Washington et al., 2014).

With the recent increase in online social interactions, the potential use of group contingency (GC) has been explored as a technology-based strategy (Dallery et al., 2019). GC is a way of classifying multiple people into one group and applying the same target behavior, criterion for delivering reinforcement, and reinforcers to all group members. It is effective, because it encourages desirable behavioral changes by promoting positive social interactions, such as praise and cooperation, among group members (Fabiano and Pelham, 2003; Popkin and Skinner, 2003). It is also more efficient than individual contingency, because the same CM can be applied at the group level, thereby decreasing the cost and effort of the manager (Herman and Tramontana, 1971; Gresham and Gresham, 1982; Deshais et al., 2019). Three types of GC have been identified in the literature (Litoe and Pumroy, 1975): independent, where the reinforcer is provided to each team member who has met the target; interdependent, where the reinforcer is provided to all team members when all of them have met the target; and dependent, where the reinforcer is provided to all team members when one or more chosen team member(s) have met the target (Litoe and Pumroy, 1975).

Although research on technology-based GC is scarce, studies in this area have suggested that technology-based GC is an effective and cost-efficient strategy. For example, in a study that aims to promote smoking cessation, 32 smokers were divided into teams of 2–3 people each, and a factorial design study with online discussion forum as the between-subjects factor and GC (independent GC, interdependent GC, and control groups) as the within-subject factor was conducted. The authors found that the interdependent GC group showed effectiveness similar to that of the independent GC group for smoking abstinence but that it was more cost efficient regardless of whether participants had access to the online discussion forum (Meredith and Dallery, 2013). In another study, 43 smokers were divided into teams of 2–3 people each, and the teams were randomly assigned to an interdependent GC group or a group that combined independent and interdependent GCs. The findings showed that, although both groups were equally effective for smoking abstinence, interdependent GC was more cost-efficient than combined GC (Dallery et al., 2015b). Furthermore, these studies demonstrate that, like traditional GC, technology-based GC is effective at promoting positive social interactions among participants (Dallery et al., 2019). The results of prior studies, which show that social factors such as social interaction and social support promote physical activity (Duncan et al., 2005; Chaudhury et al., 2016), suggest that technology-based GC might also be effective at promoting physical activity.

To date, however, there has only been one study that applied technology-based GC to promote physical activity. In this study, 304 workers were grouped in teams of 3–4 members each and were randomly assigned to one of the following three conditions: interdependent GC, a combination of interdependent and independent GC, and control (Patel et al., 2016a). The results showed that the combination condition was the most effective at promoting walking, suggesting feasibility of technology-based GCs in promoting physical activity. Nonetheless, additional research is needed to establish its effectiveness and cost-efficiency. First, before applying technology-based GC, the relative effectiveness of the three types of GC should be directly compared to identify the most effective type (Vargo and Becknell, 2019). Second, to determine whether technology-based CM is suitable for a large user base, it will be necessary to obtain information about its cost-efficiency in order to maximize the effects on behavioral change relative to costs, such as financial and human resources (Petry and Simcic, 2002; Dallery et al., 2015a).

This study aimed to determine the effects of technology-based GC applied *via* mobile devices on the promotion of walking among adults, and to verify whether there are differences in effectiveness and cost efficiency between the different types of GC.

## Materials and Methods

### Research Participants

**Figure 1:**
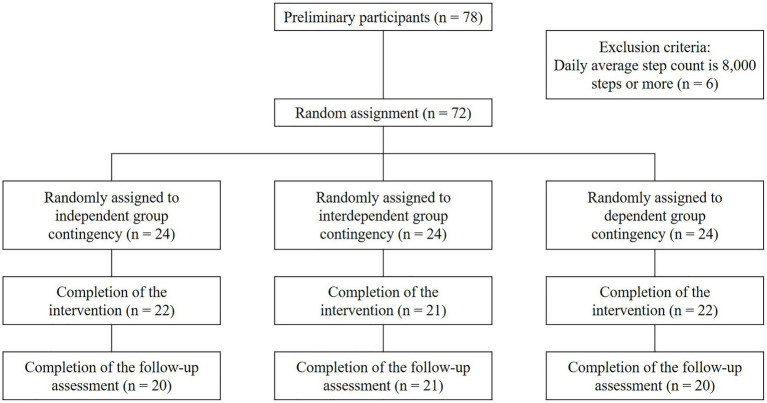
Flowchart of the participant selection process.

In this study, the participants were undergraduate and graduate students attending one of 11 universities located in the Seoul metropolitan area. The participants were recruited through advertisements on the online community of each university and *via* e-mail. A total of 78 participants (males = 29; females = 49) were recruited, and they attended the orientation and pre-intervention assessment. Of these participants, six were excluded because their mean daily step count during the baseline period was over 8,000 steps, which was set as the exclusion criteria for this study since it is considered sufficient walking (Tudor-Locke et al., 2002). The remaining 72 participants (males = 27; females = 45) were included in the intervention. Of these participants, 65 (mean age = 25 years; SD = 3.35; males = 24, females = 41) completed the intervention, and 61 (males = 22, females = 39) completed the follow-up assessment conducted 2 months after the intervention. Figure 1 shows the participant selection process. All participants who completed the post-intervention assessment received US$ 80 as compensation, and those who completed the follow-up assessment received an additional US$ 10. This study was conducted as part of a government-funded research project. The research methodology and participant recruitment procedure were similar to those of other studies with the same research project (Lee, 2019; Cho and Chung, 2020). This study was approved by the Yonsei University Institutional Review Board (IRB approval no: 7001988-202003-HR-591-09).

### Measures

#### Mobile Application “Pacer”

Pacer^1^ was used to measure step counts of the participants. It is a health management mobile application with a pedometer function that can be downloaded for free from Google Play Store or the iOS Store. Pacer runs automatically in the background of the operating system of a smartphone and measures step counts even when a user is not directly using the application. In this study, a paid administrator service was used to export the data of step count of the participants to Microsoft Excel files.^2^

#### Korean Version of International Physical Activity Questionnaire-Short Form

Korean version of the International Physical Activity Questionnaire Short Form (IPAQ-SF; IPAQ Research Committee, 2005), developed by the WHO and validated by Oh et al. (2007), was used to measure the level of walking activity of the participants. IPAQ-SF consists of seven questions. There are two questions each (duration per day and number of days per week) about three major types of activity (vigorous, moderate, and walking), and one question about time spent sitting. In this study, only the two questions about walking activity were used. Following the IPAQ-SF scoring protocol (IPAQ Research Committee, 2005), walking activity during the previous 7 days was calculated in terms of metabolic equivalents of task (MET-min/week), which is a measure of energy consumption.^3^ The MET-min/week of walking activity was calculated using the following equation: “3.3 (MET level)” × “duration of activity (min)” × “number of days of activity (days).” A higher score indicates a higher level of walking activity.

#### Physical Activity Group Environment Questionnaire

To assess the perceived group cohesion in participants during the intervention, the Physical Activity Group Environment Questionnaire (PAGEQ), developed by Estabrooks and Carron (2000), was administered for post-intervention assessment. In this study, to use the scale, it was translated using a three-step translation process (Brislin, 1970). First, a Korean-English bilingual researcher translated each item into Korean, while another researcher whose native language is English back-translated the items. Next, a clinical psychologist reviewed the validity of the translation. Finally, a scholar of PhD in Korean literature edited the text. Expressions that were ambiguous or difficult to understand were clarified and revised through discussion among the researchers. In accordance with the purpose of this study, the word “physical activity” in PAGEQ was replaced with “walking activity.” Each item was rated on a nine-point Likert scale, ranging from 1 (very strongly disagree) to 9 (very strongly agree). A higher score indicates that a participant feels attracted to the group task and social activity, and that he/she perceives unity among the team members. The internal consistency coefficients (Cronbach’s α) of the subscales were 0.91, 0.87, 0.72, and 0.85, as reported by Estabrooks and Carron (2000); and 0.9, 0.93, 0.91, and 0.64, for this study.

### Intervention Satisfaction and Utility Assessment Questionnaire

To evaluate perceived intervention satisfaction and utility in the participants, the Intervention Satisfaction and Utility Assessment Questionnaire (ISUAQ) developed by Lee (2019) was used in the post-intervention assessment. This questionnaire comprises four items: two items measuring the satisfaction of participants with target step counts and points awarded, and two items measuring the usefulness of target step counts and points to promote walking. The items about satisfaction were rated on a five-point Likert scale from 1 (very dissatisfied) to 5 (very satisfied), and the items about utility were rated on a five-point Likert scale from 1 (not at all useful) to 5 (very useful). A higher score indicates that a participant perceived greater intervention satisfaction and utility.

### Procedure

This study was conducted in the following order: orientation and pre-intervention assessment, baseline technology-based GC intervention, post-intervention assessment, and follow-up assessment.

#### Orientation and Pre-intervention Assessment

Seventy eight participants took part in the orientation and pre-intervention assessment. The orientation was conducted in person and in small groups in which the research procedure and purpose were described and the necessary pre-intervention procedures were performed. The orientation was conducted by two researchers who are MA-level graduate students in clinical psychology. The participants arrived at the prearranged time for orientation. The researchers described the procedure. Those who agreed to participate in the research were asked to sign a consent form. The participants were then told that virtual “points” would be awarded based on their step counts during the intervention, and that the total number of points could be cashed out at a ratio of 1 cent per 10 points, up to a maximum of US$80, at the end of the study. Next, the participants were instructed to download Pacer to their personal smartphones to track their step counts. In order to control the effect of other functions embedded in Pacer on the walking behavior of the participants, all alarms and feedback functions on Pacer were disabled. Furthermore, the participants were instructed to register to a group on Pacer created by the researchers so that the researchers can access step count data of the participants through the administrator service of Pacer. Finally, in order to remotely deliver messages to the participants throughout the research period, the participants were instructed to add the “KakaoTalk channel” account created by the research team on their personal KakaoTalk account.^4^ As a marketing platform for users of the online messenger KakaoTalk, a KakaoTalk channel^5^ is able to send group messages through linkage with an external website.

After the orientation, IPAQ-SF was administered through Qualtrics,^6^ an online questionnaire system, to examine the level of walking activity of the participants before the intervention. The reasons for using both Pacer and IPAQ-SF were as follows: (1) past studies recommend using different domain assessment tools in physical activity studies (Troiano et al., 2012; Dall et al., 2017; Lines et al., 2020). (2) Using different domain assessment tools is also recommended in experimental psychology.

#### Baseline

To divide the participants into teams and set a target step count appropriate for the current level of walking for each team, the step counts of the participants were measured during a 1-week baseline period (5 weekdays and weekend). To control for possible changes in the step counts due to participation in the study, the participants were instructed to continue with their usual routine until they receive a notice for the start of the experiment. Based on a previous report (Tudor-Locke et al., 2002) that suggests an average daily step count of more than 8,000 steps is sufficient to satisfy United States public health guidelines (Pate et al., 1995), six participants with an average daily step count of over 8,000 steps were excluded from the intervention. This criterion was applied to select participants who needed to increase their step count to prevent problems caused by physical inactivity, such as diabetes, obesity, and heart disease.

The participants were then divided into teams based on the daily average step count measured during the baseline period, and a target step count was set for each team. A total of 24 teams were made, with each team comprising three participants with similar average daily step counts during the baseline period. The following three measures were taken to minimize step count differences among participants assigned to the same team. First, in accordance with the criteria suggested in previous research (Kamada et al., 2018; Tomata et al., 2019), all participants were divided into the following four categories based on their average daily step count: below 2,000; 2,000–3,999; 4,000–5,999; and 6,000–7,999 steps. Second, participants in the same category were randomly grouped to form teams with three members each. Third, participants who were not assigned to a specific team in the second step were grouped into teams of three to ensure that the differences in average daily step counts among members did not exceed 2,000 steps. Of the 24 teams generated through this process, there was no team in which the differences exceeded 2,000 steps.

Three steps were then implemented to establish a suitable target step count for each team. First, the baseline average daily step count of each team was calculated. Second, based on previous findings that increasing the average daily step count by 3,000 steps is effective in promoting physical activity (Wilde et al., 2001; Tudor-Locke et al., 2005), the target step count of each team was set 3,000 steps higher than the baseline of a team. However, because goals that increased incrementally are more effective at facilitating behavioral change than goals that increased at once (Cooper et al., 2020), each team’s target step count was increased 1,500 steps each only in the first two out of three periods, then set the same as the second period during the last period (Baker et al., 2008). Third, based on the prior finding that increasing time interval to meet a goal could reduce the burden to achieve it (Kurti and Dallery, 2013), the time interval to meet the target step count was set to 3 days. The final target step counts during the intervention are shown in Table 1.

**Table 1:** Target step count during the intervention period.

Type	Duration (interval number)	Target step count per 3-day interval
Increasing	Days 1–21 (eight intervals)	(Baseline average daily step count of the team + 1,500 steps) × 3 (days)
Increasing	Days 22–42 (seven intervals)	(Baseline average daily step count of the team + 3,000 steps) × 3 (days)
Maintenance	Days 43–66 (eight intervals)	(Baseline average daily step count of the team + 3,000 steps) × 3 (days)

The KakaoTalk-Open Chat^7^ service was used to provide an anonymous online chat room for each team to enable social interaction among team members. Previous studies that examined technology-based GC also used online channels for interactions among participants (Meredith et al., 2011; Meredith and Dallery, 2013; Dallery et al., 2015b), since social interaction is considered essential in GC (Gresham and Gresham, 1982; Williamson et al., 1992). KakaoTalk-Open Chat is a supplementary service offered by the online messenger KakaoTalk. It supports an anonymous online chat room in which individuals can participate without disclosing their profile or personal information. It also offers the option for disabling access to people outside a defined set of users by specifying a participation code. The participants were informed that they could freely send and receive messages among the invited participants in the anonymous chat room, and that the researcher would moderate the chat in case of any profanity or slander. However, profanity or slander in the chats was not observed during the research period.

#### Intervention

**Figure 2:**
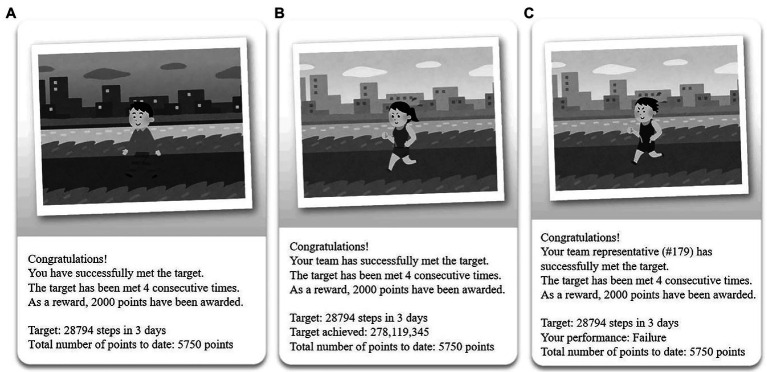
Example outcome message for each condition: **(A)** Independent group contingency (GC), **(B)** Interdependent group contingency, and **(C)** Dependent group contingency.

Before the intervention, the 24 teams were randomly assigned to one of the following three conditions: independent GC, interdependent GC, or dependent GC. All teams in the three conditions received the intervention for 66 days (a total of 22 3-day intervals; Lally et al., 2010). Points were given to participants who met the target step counts and increased if the target step counts were met consecutively. The points were initially set at 1,000 per 3-day interval, with a rate of 250 points for each consecutive success. These values were set based on the study of Cho and Chung (2020) on point systems. The total maximum points that a participant could obtain upon meeting a target step count is 79,750 points, regardless of conditions [e.g., interval 1 (1,000) + interval 2 (1,000 + 250) + interval 3 (1,000 + 250 + 250) + … + interval 22 (1,000 + 250 + 250 + … + 250) = 79,750]. At 2 pm on the day following a 3-day interval, all the participants received a text message with an image,^8^ which was generated *via* the following steps: first, the step counts of the participants, measured through Pacer, were uploaded to the custom-developed personal hypertext preprocessor (PHP)-based administrative website.^9^ Second, the algorithm embedded on the website would automatically decide whether the target step count was achieved or not for each participant. Third, a message was automatically sent to each participant through the KakaoTalk channel. Each message included information about the target step count of the participants and whether the target was met, the number of consecutive times the target step count was met, the number of points obtained in a 3-day interval, and the most recent total number of points obtained (Figure 2).

##### Independent GC

The participants under the independent GC condition received points if they met the target step count, regardless of the performance of other team members. The initial points were 1,000, and were increased by 250 points per 3-day interval if the target step counts were met consecutively. The points were reset to the initial ones if the target step counts were not met.

##### Interdependent GC

The participants under the interdependent GC condition received points if all team members met the target step count. If one or more team member(s) failed to meet the target step count, none of the team members received any points for a particular 3-day interval. The initial points, increasing point system, and reset rules were exactly the same as those for the independent GC. Information about performance among team members was also provided in the message.

##### Dependent GC

The participants under the dependent GC condition received points if a designated team member met the target step count. If the designated team member failed to meet the target step count, none of the team members received any points for a particular 3-day interval. For each 3-day interval, a team member was randomly selected to represent the team for that particular interval by an algorithm on the website set by the researchers prior to the experiment. Each team member had an equal chance of being selected. However, no information was provided to the team members regarding who was selected until the end of the 3-day interval. The initial points, increasing point system, and reset rules were exactly the same as those for the other two groups. Information about the team representative for each 3-day interval was also provided in the message.

#### Post-intervention Assessment

Within 5 days from the termination of the intervention, all participants revisited the laboratory to undergo a post-intervention assessment in which questionnaires same as those in the pre-intervention assessment were administered, along with the PAGEQ and the ISUAQ. After completion of the post-intervention assessment, a debriefing form was provided, which included the reinforcement criteria for each condition, experimental procedure, and compensation formula. All participants who completed the post-intervention assessment could earn US$80 as compensation.

#### Follow-Up Assessment

To examine the maintenance of the intervention effect, a follow-up assessment was conducted 2 months after the completion of the experiment. The follow-up assessment was optional and only included those who agreed to participate in advance. The step count data of the participants were collected *via* Pacer during the 2-month follow-up period, and questionnaires same as those in the pre-and post-intervention assessments were administered. All participants who completed the follow-up assessment received US$10 as compensation.

### Data Analysis

An *a priori* power analysis was performed (G*Power; Faul et al., 2007) for a repeated measures ANOVA of between–within interactions with a three-group design by setting *α* error probability to 0.05 and 1-*β* error probability to 0.90. The effect size *F* was set to 0.25 (Cohen, 1988), and correlation among repeated measures was set to 0.3 based on the results of previous studies (Lee, 2019; Cho and Chung, 2020). As a result, the minimum sample size was found to be 60 participants. Based on this result and predicted drop-out rates, data were collected from a total of 72 participants.

IBM SPSS (Statistical Package for the Social Sciences) Windows Version 25.0 was used for statistical analyses. For variables that satisfied the normality assumption according to the Shapiro-Wilk test (step counts, walking activity, and total scores in PAGEQ), parametric tests (one-way ANOVA and two-way mixed model repeated-measures ANOVA) were performed. For variables that did not satisfy the normality assumption (target step count was met, increase in step count per point, increase in level of walking activity per point, and intervention satisfaction and utility scores in ISUAQ), nonparametric tests [generalized estimating equation (GEE) and the Kruskal–Wallis H-test] were performed. To assess for homogeneity among the conditions before the intervention, a chi-square test was performed on sex, and one-way ANOVA was performed on the pre-intervention step counts and walking activity. The analysis revealed no significant difference in sex ratio, step counts, and walking activity by the GC type.

First, to determine the effects of technology-based GC and compare the effectiveness of the three conditions, two analyses were performed as follows: GEE was performed to analyze the interaction effects of condition (independent GC, interdependent GC, and dependent GC) × time (22 3-day intervals). The dependent variables were binary and indicated whether the target step count was achieved during a 3-day interval (success: 1, failure: 0). To compensate for the shortcomings of other analyses that completely exclude missing data, GEE used various missing data processing methods (Robins et al., 1995). One of these methods was used to estimate the missing data based on previous measurements and include them in the analysis (Diggle et al., 2002; Qu and Song, 2002). Therefore, for the GEE analysis only, the last observation carried forward was used to utilize data from 72 participants, including those who dropped out. Following this, whether the target step count was achieved by the participant who dropped out was imputed by carrying forward the last measured observation (Womble et al., 2004). The second analysis, a two-way mixed model repeated measures ANOVA, was performed to analyze the interaction effects of condition (independent GC, interdependent GC, or dependent GC) × time (pre-intervention, post-intervention, and follow-up). The dependent variables were step counts and walking activity of the 61 participants who completed the intervention and the follow-up assessment. Unlike GEE, the two-way mixed model repeated measures ANOVA included follow-up data.

Second, to compare the cost efficiency of the three conditions, the Kruskal–Wallis H-test was performed on the 65 participants who completed the intervention. The dependent variable was an increase in step count and in the level of walking activity per point in each condition. The increase per point was calculated with the following equation: post-intervention measurement-pre-intervention measurement/total number of points obtained.

Third, to examine whether there were differences in group cohesion and social validity by the GC type, one-way ANOVA and the Kruskal–Wallis H-test were performed on the 65 participants who completed the intervention. Dependent variables were the total scores in PAGEQ and the intervention satisfaction and utility scores in ISUAQ.

## Results

### Effectiveness of the Intervention

**Figure 3:**
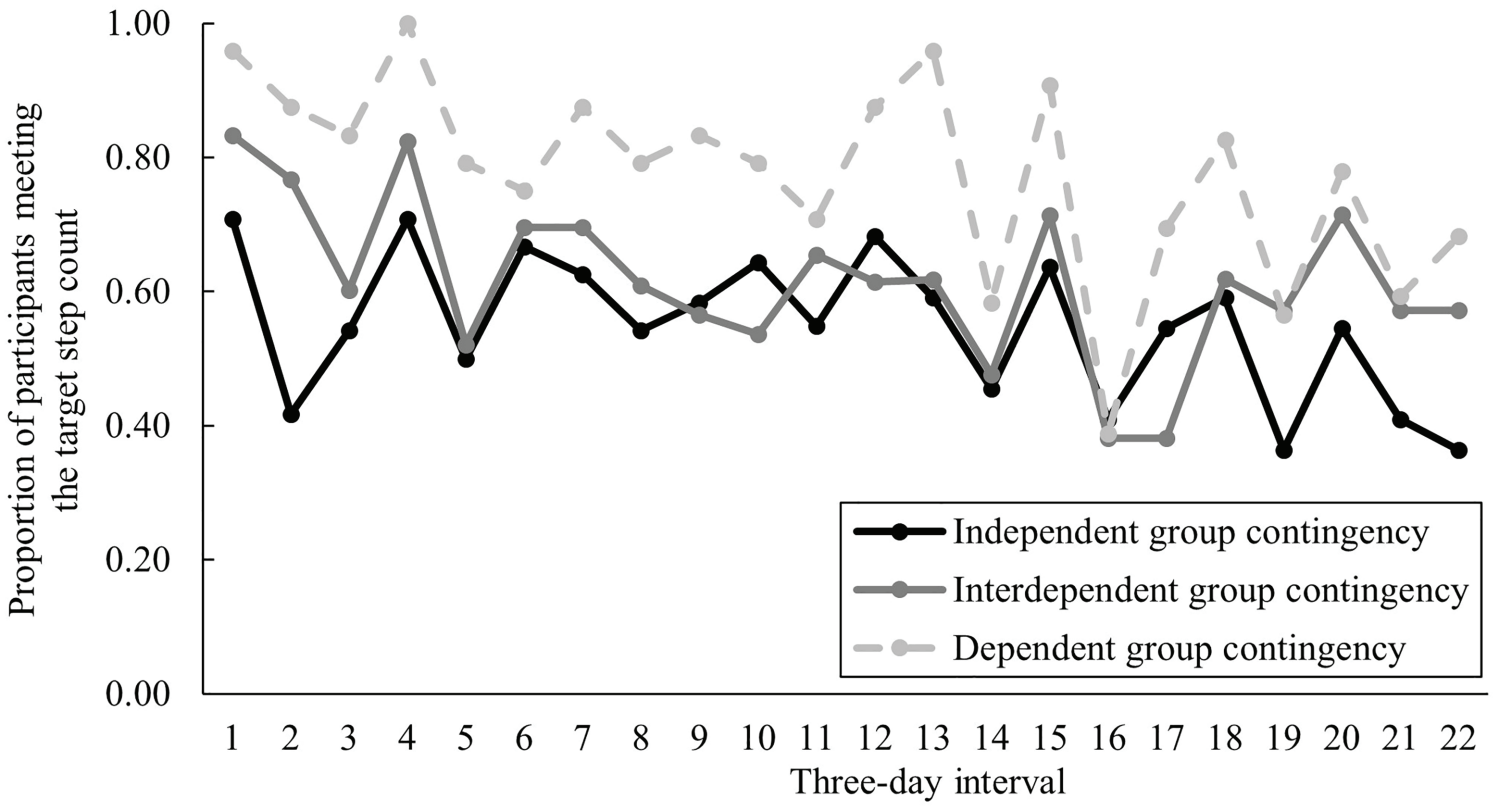
Changes in the proportion of participants meeting the target step count in each 3-day interval by condition.

To examine effectiveness, first, GEE was performed with success/failure (Success: 1, Failure: 0) to meet the 22 target step count (*N* = 72) as the dependent variable. An analysis showed that the interaction between condition and time was significant (Table 2), indicating that there were differences in the proportion of participants who meet the target step count among the three conditions (Figure 3). Specifically, the success rate was higher under the dependent GC condition than that under the independent and interdependent GC conditions across all times. As comparing changes in goal attainment over time was not the purpose of this study, no additional *post hoc* analysis was performed on the time factor. The main effects of condition and time were also significant. The Bonferroni correction results showed that the success rate of meeting the target step count during the intervention under the dependent GC condition was significantly greater [0.93 (95% CI 0.89, 0.96)] than that under the independent GC [0.55 (95% CI, 0.42, 0.68), *p* < 0.001] and interdependent GC conditions [0.62 (95% CI 0.5, 0.73), *p* < 0.001].

**Table 2:** Results of generalized estimating equation (GEE) on whether the target step count was met during the intervention.

	Wald chi-square	*Df*	*p*
Condition	44.07	2	0
Time	1,214.74	21	0
Condition × Time	5,581.87	41	0

Second, two-way mixed model repeated measures ANOVA was performed with step counts and walking activity (each *N* = 61) as dependent variables. An analysis of the step counts showed that the two-way interaction between condition and time was significant (Table 3). However, the Bonferroni correction results showed no differences between the conditions in the pre-intervention, post-intervention, and follow-up assessments (*p* > 0.05). Moreover, the main effect of the condition was not significant, but that of time was. The results of the Bonferroni correction showed that the post-intervention step counts were significantly higher than the pre-intervention (*p* < 0.001) and follow-up (*p* < 0.001) step counts. A similar analysis of walking activity showed that the two-way interaction between condition and time was not significant (Table 3). Similarly, the main effect of the condition was not significant, but that of time was. The Bonferroni correction results showed that the level of post-intervention walking activity was significantly higher than that of pre-intervention walking activity (*p* < 0.05), but there was no significant difference compared with that of the follow-up walking activity (*p* > 0.05). The means and SDs of the pre-intervention, post-intervention, and follow-up step counts and walking activity by the condition are presented in Table 4.

**Table 3:** Results of two-way mixed model repeated measures ANOVA on step counts and walking activity.

Source	Sum of squares	*df*	Mean square	*F*	*p*	Partial *η*^2^	*Post hoc*
**Step counts**							
Intercept	8,141,103,974.22	1	8,141,103,974.22	1,469.79	0	0.96	
Condition	10,331,424.13	2	5,165,712.06	0.93	0.4	0.03	
Time	274,014,341.33	1	274,014,341.33	248.08	0	0.81	Post > pre, fu^*^
Condition × time	7,392,703.62	2	3,696,351.81	3.35	0.04	0.1	
Error (time)	64,064,191.14	58	1,104,555.02				
Error	321,258,667.19	58	5,538,942.54				
**Walking activity**							
Intercept	116,688,711.49	1	116,688,711.49	140.49	0	0.77	
Condition	1,166,275.54	2	583,137.77	0.7	0.5	0.03	
Time	1,149,028.51	1	1,149,028.5	4.51	0.04	0.1	Post > pre^*^
Condition × time	333,818.17	2	166,909.08	0.65	0.53	0.03	
Error (time)	10,964,180.62	43	254,980.94				
Error	35,714,135.03	43	830,561.28				

* *p* < 0.05.

**Table 4:** Pre-intervention, post-intervention, and follow-up measurements of walking by condition among participants who completed follow-up.

Variable		Independent group contingency	Interdependent group contingency	Dependent group contingency
	*N*	20	21	20
Step counts	Pre *M* (*SD*)	5,575.56 (1,457.86)	5,778.93 (1,160.6)	5,637.17 (1,508.65)
	Post *M* (*SD*)	7,884.45 (2,296.14)	8,218.04 (1,294.9)	9,105.28 (1,151.68)
	Fu *M* (*SD*)	5,696.15 (2,656.75)	5,976.21 (2,177.24)	6,172.7 (1,818.94)
	*N*	13	16	17
Walking activity	Pre *M* (*SD*)	844.04 (576.39)	614.63 (314.48)	741.53 (483.71)
	Post *M* (*SD*)	1,035.69 (754.51)	961.13 (729.18)	1,169.75 (707.61)
	Fu *M* (*SD*)	1,139.77 (852.02)	814.69 (512.36)	1,009.41 (944.28)

### Cost Efficiency of the Intervention

To examine cost efficiency, the Kruskal–Wallis H-test was performed with an increase in step count per point (*N* = 65) and an increase in the level of walking activity per point (*N* = 65) as dependent variables. The analysis of the increase in step count per point showed that there were significant differences among the conditions [*H*(2) = 14.75, *p* < 0.01]. The Bonferroni correction results showed that the step count increase per point under the interdependent GC condition was significantly higher than that under the independent and dependent GC conditions (both *p* < 0.01). Next, the analysis of the increase in the level of walking activity per point showed that there were no significant differences between the conditions [*H*(2) = 2.37, *p* > 0.05]. The means and SDs of the variables related to cost efficiency by the condition are presented in Table 5.

**Table 5:** Measurements of variables related to cost efficiency by condition.

Variable		Independent group contingency	Interdependent group contingency	Dependent group contingency
	*N*	22	21	22
Increase in step count per point	*M* (*SD*)	0.01 (0.32)	0.54 (0.67)	0.11 (0.06)
	*n*	17	17	18
Increase in walking activity per point	*M* (*SD*)	0 (0.14)	0.05 (0.23)	0.01 (0.03)

### Group Cohesion and Social Validity of the Intervention

To examine group cohesion, one-way ANOVA was performed with the total scores in PAGEQ (*N* = 65) as dependent variables. The analysis showed significant differences among conditions [*F*(2, 62) = 5.93, *p* < 0.01]. The Bonferroni correction results showed that the group cohesion score under the dependent GC condition was significantly higher than that under the independent GC condition (*p* < 0.01).

Additionally, to examine social validity, the Kruskal–Wallis H-test was performed with the intervention satisfaction and utility scores in ISUAQ as dependent variables (each *N* = 65). The analysis showed that there were significant differences in point satisfaction and point utility between the conditions [*H*(2) = 8.9, *p* < 0.05 and *H*(2) = 7.99, *p* < 0.05, respectively]. The Bonferroni correction results on each subscale showed that point satisfaction and point utility under the dependent GC condition were significantly higher than those under the interdependent GC condition (both at *p* < 0.05). There were no significant differences in target satisfaction and target utility between the conditions [*H*(2) = 4.35, *p* > 0.05 and *H*(2) = 5.31, *p* > 0.05, respectively]. The means and SDs of the variables related to group cohesion and social validity by the condition are presented in Table 6.

**Table 6:** Measurements of variables related to group cohesion and social validity by condition.

Variable			Independent group contingency	Interdependent group contingency	Dependent group contingency
		*N*	22	21	22
Group cohesion	Total score in PAGEQ	*M* (*SD*)	56.05 (24.78)	70.33 (33.12)	86.69 (29.97)
		*N*	22	21	22
Social validity	Target satisfaction	*M* (*SD*)	3.32 (1)	3.38 (−1.02)	3.91 (−0.97)
	Target utility	*M* (*SD*)	3.5 (−1.06)	3.57 (−1.25)	4.14 (−0.89)
	Point satisfaction	*M* (*SD*)	3.59 (−0.91)	2.95 (−1.07)	3.82 (−1.05)
	Point utility	*M* (*SD*)	3.5 (−1.41)	3.14 (−1.19)	4.09 (−1.19)

## Discussion

This study measured the effect of technology-based GC, applied using a mobile device, on the promotion of walking among adults, and sought to compare the effectiveness and cost efficiency between different types of GC.

In terms of effectiveness, the study found technology-based GC *via* mobile devices to be an effective strategy to promote walking among adults, regardless of the GC type. In all the GC conditions, the step counts at post-intervention increased by approximately 2,700 steps (48.35%), compared with the baseline. These results are consistent with the findings from several traditional, face-to-face studies (Vidoni et al., 2014; Kuhl et al., 2015; Foote et al., 2017), and recent studies demonstrating the effectiveness of technology-based GC to promote physical activity (Patel et al., 2016a). In GC, participants are classified into a group where target behavior, reinforcement criterion, and reinforcers are all identical. Since the consequences (e.g., points given to participants in this study) are decided based on the performance of each group member, it is assumed that GC facilitates peer pressure and positive social interactions, such as praise and cooperation among group members (Fabiano and Pelham, 2003; Popkin and Skinner, 2003). Accordingly, few researchers have proposed that social support is the primary mechanism for behavioral change in GC (Meredith and Dallery, 2013; Dallery et al., 2015b, 2019; Cooper et al., 2020). The positive findings of this study also indirectly support the presence of a similar mechanism within a technology-based GC, wherein online interactions *via* mobile devices influenced behavioral change.

Among the three GC conditions, dependent GC was found to be the most effective. The results showed that the average rate to achieve the target step counts in the dependent GC condition (0.93) was approximately 1.5 times the average rate observed in the independent and interdependent GC conditions (0.55 and 0.62, respectively). The increased effectiveness of dependent GC may be attributed to the level of responsibility or peer pressure that each team member might have experienced during the intervention (Theodore et al., 2004; Williamson et al., 2009). That is, in the dependent GC condition, a team representative was randomly selected during a 3-day interval, but the team members were not notified of this until the end of the interval. Therefore, each participant is likely to have experienced a sense of responsibility and/or peer pressure to achieve the target step count, since the reward for their group could have been dependent on their performance. On the other hand, in the independent GC condition, the performance of a participant did not affect the rewards received by the other team members, or vice versa. Similarly, in the interdependent GC condition, the rewards were given only when all the team members achieved the target step count, thereby placing equal responsibility on all members. A higher level of perceived responsibility in the dependent GC condition has also been reported in previous literature (Scott et al., 2017). However, further investigation is necessary as this study does not measure other factors that may have affected participation in a group intervention, such as personality traits.

Furthermore, within the dependent GC condition, random selection ensures that the likelihood of each member being selected as team representative is equal, thereby preventing any criticism or blame on a particular team member when the reward is not obtained (Gresham and Gresham, 1982; Heering and Wilder, 2006; Williamson et al., 2009; Cariveau and Kodak, 2017). In fact, the results of this study showed that group cohesion and satisfaction with the intervention were higher under the dependent GC condition. This supports previous claims that random selection motivates engagement in the target behavior by enhancing the social validity of the intervention (Williamson et al., 2009). The impact of random selection on satisfaction and effectiveness of the intervention must be systematically explored through future research.

In terms of cost efficiency, interdependent GC was proven to be the most cost-efficient. Specifically, the increase in step count per 1,000 points was 540 steps under the interdependent GC condition, as opposed to 110 steps under the dependent GC condition and 10 steps under the independent GC condition. This finding is consistent with that of previous studies that used technology-based GC to promote smoking cessation (Meredith and Dallery, 2013; Dallery et al., 2015b). Compared with other GC types, the higher cost efficiency of the interdependent GC condition seems to be related to the difficulty in receiving the reward. The percentage of participants who received rewards for goal attainment was 100% in the case of independent GC, 86% for the dependent GC condition, and 63% for the interdependent condition. This is attributable to the nature of interdependent GC, which requires all participants to meet the target without any exceptions. However, under this condition, points were rewarded relatively infrequently. The average monetary reward per participant during the 66 days under the interdependent GC condition was approximately 40% lower (US$15.25) than that under the dependent GC condition (US$40.14). As a result of this difference in reward, the interdependent GC was found to be an efficient strategy for facilitating the physical activity. However, given the superiority of dependent GC in terms of effectiveness, and the importance of participants maintaining satisfaction in technology-based CM (Raiff et al., 2013), the low level of user satisfaction and utility reported in the interdependent GC condition might negatively affect continuous and consistent engagement with the intervention program. Therefore, it is necessary to examine the various factors involved in different GC conditions when choosing the most appropriate strategy.

In terms of the maintenance effect of the intervention, step counts of participants regressed to the baseline level at follow-up. Such decrease in behavior following the withdrawal of reinforcement has been reported in previous research that examined the effectiveness of GC (Patel et al., 2016a; Foote et al., 2017) or other types of CM in promoting physical activity (Finkelstein et al., 2008; Patel et al., 2016b, 2018; Weinstock et al., 2016). In contrast, some other studies, which have also examined the effectiveness of CM in promoting physical activity (Hunter et al., 2013; Petry et al., 2013) or drug discontinuation (Epstein et al., 2003; García-Fernández et al., 2011; Secades-Villa et al., 2011; Petitjean et al., 2014), have reported sustained effects even months after termination of the intervention. There have been several possibilities speculated upon for these differences. For instance, a possibility is that the intervention period was not long enough. In this study, the intervention lasted for 66 days, which was previously reported to be the minimum duration necessary to change habits (Lally et al., 2010). However, the general principle of behavioral change states that a more detailed reinforcement schedule is necessary to change a behavior that already occurs frequently in daily life, such as walking (Cooper et al., 2020). In view of this, the intervention may need to be longer than 66 days for its effect to be maintained. Indeed, studies that reported a sustained effect even after the withdrawal of reinforcement conducted interventions that lasted 84 days, a duration that is approximately 20 days longer than that used in this study (Hunter et al., 2013; Petry et al., 2013). Another possible explanation is the difference in the kinds of behavioral change strategies used. Previous research has reported that a combination of multiple strategies is more effective than a single strategy (Watson et al., 2012; Patel et al., 2018). Indeed, long-term effects have been reported when CM was combined with face-to-face counseling (García-Fernández et al., 2011; Secades-Villa et al., 2011) or cognitive behavioral therapy (Epstein et al., 2003; Petitjean et al., 2014). Further, while these studies focused on drug discontinuation, they also suggested additional strategies that could be helpful for promoting physical activity.

Additionally, this study highlighted the inaccuracy of reports of physical activity gathered using self-report questionnaires. In other words, during the follow-up period, a significant decrease was observed only for the device-based measure (step counts) but not for the self-reported measure (self-reports on walking activity). This is consistent with previous research that reported similar differences (Tucker et al., 2011; Siebeling et al., 2012). There are some possible explanations for these differences. First, it is possible that the participants overestimated their level of physical activity in an attempt to give socially desirable responses (Hebert et al., 1997; Sallis and Saelens, 2000). Prior studies have reported that this tendency could be induced by intervention procedures with a specific goal (Taber et al., 2009). Second, the differences might be attributable to the characteristics of the IPAQ-SF items used in this study. The IPAQ-SF calculates walking activity by asking the respondent to recall the number of days, within the last 1 week, on which they walked for 10 min or more and the average duration of time spent walking per day. Therefore, it is possible that the distortion in responses could have arisen because of the difficulty of the recall process. In particular, given that walking is a behavior that occurs in most situations, regardless of location or time, distortion during recall can occur relatively easily.

In conclusion, the findings in this study have few implications on the development process used for mHealth applications in the future. First, the findings offer crucial information regarding the usability of different GC conditions and their challenges. For instance, when applying technology-based GC to an mHealth system with the goal of effective promotion of physical activity, the dependent GC could be the first choice, along with the adaptation of other components, such as online chatting and random selection of a team representative in order to encourage group cohesion. Similarly, an interdependent GC could be utilized in case of any financial limitations. However, it is important to consider not only cost efficiency but also other factors, such as effectiveness and user satisfaction. Second, when building an mHealth application, usage of device-based measures of physical activities is recommended (Sallis and Saelens, 2000; Kurti and Dallery, 2013). Finally, it is recommended that device-based measures be developed and used for physical activities other than walking (e.g., sit-ups, running, etc.).

However, the findings in this study should be interpreted in light of the following limitations: first, the sample size of this study was only slightly over the minimum number required for power analysis. Future studies with larger sample sizes are needed to establish the relative effectiveness and efficiency of group contingency. Second, the effectiveness and cost efficiency of technology-based GC can differ depending on the demographic characteristics of the participants. The sample for this study included undergraduate and graduate students who were familiar with smartphone usage. Therefore, additional research needs to be conducted with different age groups. Third, the effectiveness and cost efficiency of technology-based GC could differ depending on the target behavior, especially its characteristics (e.g., individual behavior, behavior that affects others, etc.), given the nature of the GC process. Thus, the effectiveness could differ if the technology-based GC was applied to behaviors other than walking. Fourth, the goal of this study was to examine the effectiveness of group contingencies, but only direct measures of walking, such as step count and self-report questionnaire, were obtained. Additional measures (e.g., body mass index, weight, etc.) would be beneficial to examine the consequences of increased walking. Finally, since this study utilized the principle of delayed reinforcement, which includes a time gap between the performance of the target behavior and the delivery of the reinforcer, future studies should analyze the effectiveness of mHealth systems that offer immediate reinforcement for a target behavior.

## Data Availability Statement

The raw data supporting the conclusions of this article will be made available by the authors, without undue reservation.

## Ethics Statement

The studies involving human participants were reviewed and approved by Yonsei University Institutional Review Board. The patients/participants provided their written informed consent to participate in this study.

## Author Contributions

HK designed the experiments, conducted the data collection and analysis, and shared in the writing of the manuscript. This manuscript is based on Heewon’s master’s thesis, submitted at Yonsei University, Seoul, South Korea. K-MC designed and managed the experiment, and shared in the writing of the manuscript. CL and SL recruited the participants, conducted the data collection, and shared in the writing of the manuscript. All authors contributed to the article and approved the submitted version.

### Conflict of Interest

The authors declare that the research was conducted in the absence of any commercial or financial relationships that could be construed as a potential conflict of interest.

## Footnotes

^1^Introduction to Pacer and download link: https://www.mypacer.com/

^2^Introduction and registration to the administrative service for Pacer: https://www.mypacer.com/teams

^3^Link to download the IPAQ-SF and scoring protocol: www.ipaq.ki.se/

^4^KakaoTalk is a free mobile/PC messenger that provides functions that include sending text messages and emoticons, photographs and video files, and voice and video calling. “KakaoTalk” webpage: https://www.kakaocorp.com/

^5^Administrator page for “KakaoTalk Channel”: https://center-pf.kakao.com

^6^“Qualtrics” webpage: http://www.qualtrics.com

^7^Introduction to “KakaoTalk-Open Chat” service: https://open.kakao.com

^8^Up to 20 of these illustrations can be used for free as long as they are used as a part of a design and not as self-contained content. The illustrations can be downloaded at https://www.irasutoya.com/p/faq.html

^9^To gain accessibility to the website, please contact ysuniv.clinicalpsy@gmail.com.
